# Alternative splicing of pre-mRNA modulates the immune response in Holstein cattle naturally infected with *Mycobacterium avium* subsp. *paratuberculosis*

**DOI:** 10.3389/fimmu.2024.1354500

**Published:** 2024-03-01

**Authors:** Gerard Badia-Bringué, José Luis Lavín, Rosa Casais, Marta Alonso-Hearn

**Affiliations:** ^1^ Department of Animal Health, NEIKER-Basque Institute for Agricultural Research and Development, Basque Research and Technology Alliance (BRTA), Derio, Bizkaia, Spain; ^2^ Universidad del País Vasco/Euskal Herriko Unibertsitatea (UPV/EHU), Leioa, Bizkaia, Spain; ^3^ Department of Applied Mathematics, NEIKER- Basque Institute for Agricultural Research and Development, Basque Research and Technology Alliance (BRTA), Derio, Bizkaia, Spain; ^4^ Center of Animal Biotechnology, Servicio Regional de Investigación y Desarrollo Agroalimentario (SERIDA), Deva, Spain

**Keywords:** paratuberculosis, alternative splicing, chronic inflammatory diseases, autoimmune diseases, molecular mechanisms

## Abstract

Little is known about the role of alternative splicing (AS) in regulating gene expression in *Mycobacteria*-infected individuals in distinct stages of infection. Pre-mRNA AS consists of the removal of introns and the assembly of exons contained in eukaryotic genes. AS events can influence transcript stability or structure with important physiological consequences. Using RNA-Seq data from peripheral blood (PB) and ileocecal valve (ICV) samples collected from Holstein cattle with focal and diffuse paratuberculosis (PTB)-associated histopathological lesions in gut tissues and without lesions (controls), we detected differential AS profiles between the infected and control groups. Four of the identified AS events were experimentally validated by reverse transcription-digital droplet PCR (RT-ddPCR). AS events in several genes correlated with changes in gene expression. In the ICV of animals with diffuse lesions, for instance, alternatively spliced genes correlated with changes in the expression of genes involved in endocytosis, antigen processing and presentation, complement activation, and several inflammatory and autoimmune diseases in humans. Taken together, our results identified common mechanisms of AS involvement in the pathogenesis of PTB and human diseases and shed light on novel diagnostic and therapeutic interventions to control these diseases.

## 1 Introduction

Bovine paratuberculosis (PTB) (or Johne’s disease) is a granulomatous infection caused by *Mycobacterium avium* subsp. *paratuberculosis* (MAP). PTB is a granulomatous enteritis of ruminants that must be notified to the World Organization for Animal Health. Several studies have demonstrated that more than 50% of the dairy cattle herds are positive for MAP antibodies in the USA and in Europe and, therefore, PTB can be considered endemic in these areas (1, 2). The dairy industry estimated that losses due to PTB each year are US$198 million in the United States, US$364 in the European Union, US$75 million in Germany, US$56 million in France, and US$12 million in Spain (3). Infection usually occurs at an early stage of life and can remain subclinical for years. In the jejunal-ileal Peyer’s patches, MAP bacilli gain entry into the intestinal mucosa via interaction with M cells and epithelial cells (4, 5). MAP can survive within infected macrophages by inhibiting apoptosis and phagosomal acidification, and by preventing the presentation of antigens to the immune system (6). As the infection progresses, the lesions in the intestine and lymph nodes become more severe and the granulomatous infiltrate becomes diffuse, disrupting the mucosal structure (7, 8). There is evidence suggesting that MAP might act as an environmental trigger of chronic inflammatory diseases (CIDs) such as Crohn´s disease and autoimmune diseases in humans, including multiple sclerosis (MS), Type-1 diabetes mellitus (T1DM), and rheumatoid arthritis (RA) (9, 10). CIDs are a group of disorders of unclear etiology characterized by a persistent inflammation that ultimately damages the target organs and tissues (11). Several studies have demonstrated that molecular mimicry between MAP and human peptides activates responses associated with many human autoimmune diseases (12). However, the mechanisms linking MAP infection, human inflammatory and autoimmune diseases, and immune dysregulation have not been fully elucidated. A better knowledge of these pathogen-host interactions may help to develop effective strategies to block common early pathogenic steps of these diseases.

One of the major response mechanisms that influences MAP infection outcome is host gene expression (13). Transcriptomics has been shown to be a useful tool for analyzing host gene expression changes from the latent infection to clinical disease, while functional genomics has been able to trace the molecular mechanisms that link MAP infection, host genetics, and disease outcome (14, 15). In a previous study, we found that the heterozygous genotype in the cis-eQTL-rs109859270 (C/T) was associated with the upregulation of U1 small nuclear ribonucleoprotein (snRNP) mRNA expression and with an increased risk of progression to clinical PTB (16). By contrast, the most frequent homozygous genotype (C/C) maintained the U1 snRNA expression levels in a restricted range and was associated with a lower risk of infection and disease progression. These findings suggested that host genetics can significantly impact the expression of important components of the host splicing machinery and, consequently, may compromise the splicing of a subset of introns contributing to the disease outcome. In humans, many diseases are caused by point mutations that affect pre-mRNA splicing by destroying or weakening splice sites, thereby producing mRNAs that encode defective proteins or that are targets for degradation by the nonsense-mediated mRNA decay (NMD) (17, 18).

**Figure 1:**
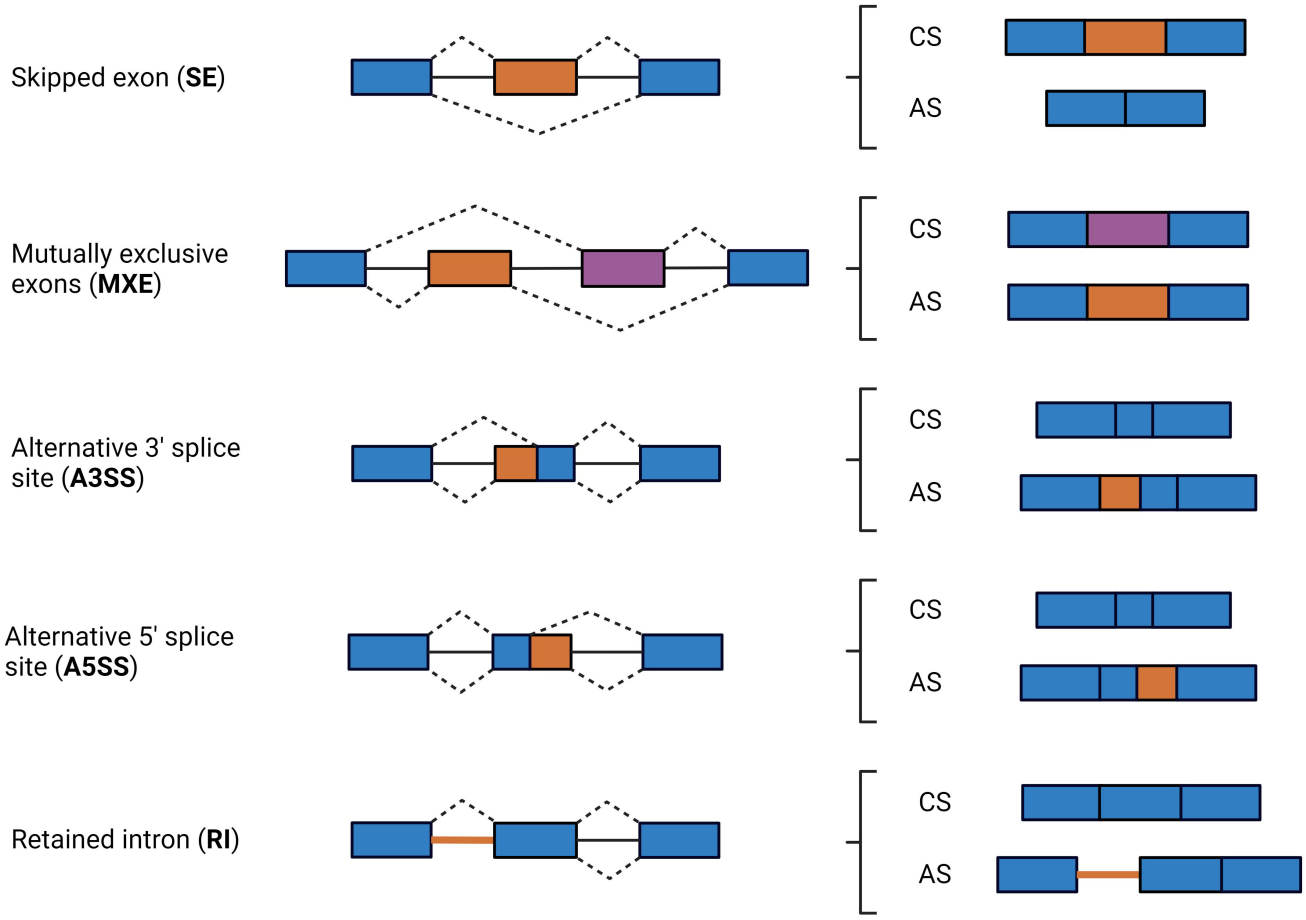
Schematic diagram of the major types of AS events. AS events are defined by the location of the splice sites in the following types: skipped exon (SE), in which a single exon is included or excluded from the final transcript; mutually exclusive exons (MXE), in which the final transcript only contains one of the two affected exons; alternative 3′ splice site (A3SS), in which the acceptor site (at 3′) is changed; alternative 5’ splice site (A5SS), in which the donor site (at 5′) is changed; retained intron (RI), an intron or a portion of an intronic region gets included in the final transcript. Exons and introns are represented by boxes and solid lines, respectively. Dashed lines indicate possible acceptor-donor combinations. Exons included in all isoforms are in blue, while exons, exon sequences, and introns that can or cannot be included by splicing events are in orange or purple. CS, constitutive splicing; AS, Alternative splicing.

For pre-mRNAs to be transported to the cytoplasm where they can direct protein synthesis, introns need to be removed. Pre-mRNA splicing consists of the removal of introns and assembly of exons contained in the eukaryotic genes by the spliceosome, a structure composed of highly dynamic snRNPs and accessory proteins, the most important one being the snRNP complex composed of U1, U2, U4, U5, and U6 snRNPs (19, 20). Spliceosome assembly starts with U1 binding to the dinucleotide GU at the 5′ splice site and the U2AF complex binding to the dinucleotide AG at the 3′ splice site. Then, U2 recognizes a key adenine called the branch point and interacts with U1 to form the pre-spliceosome. Following U4, U5, and U6 recruitment, the spliceosome enters its active conformation and proceeds to catalyze two sequential transesterification reactions that excise the intron (21). Splice sites are typically categorized as constitutive or alternative, depending on whether they are always (constitutive) or only sometimes (alternative) recognized by the spliceosome and spliced in the mature mRNA. Strong splice sites containing consensus sequences that are easily recognized by the spliceosome lead to constitutive splicing, whereas weak splice sites lead to alternative splicing (AS) and can only be recognized via the mediation of additional splicing regulatory elements. Depending on the splice site locations, AS events can be classified into different classes, including skipped exon (SE), in which a single exon is included or excluded from the final transcript; mutually exclusive exons (MXE), in which the final transcript only contains one of the two affected exons; alternative 3′ splice site (A3SS), in which the acceptor site (at 3′) is changed; alternative 5′ splice site (A5SS), in which the donor site (at 5′) is changed; and retained intron (RI), in which an intron gets included in the final transcript (**Figure 1**). AS events can have multiple detrimental consequences, one of them being the generation of premature termination codons, which are natural targets of the NMD pathway and can cause changes in gene expression (22, 23). Changes in AS events can cause disease directly, modify the severity of the disease phenotype, or be linked with disease susceptibility (24). Changes in AS events are responsible for infection, inflammation, and immune and metabolic diseases (25, 26).

Little is known about the genome-wide post-transcriptional regulation after exposure to human or animal pathogens. Recently, RNA sequencing (RNA-Seq) has become a novel powerful approach for the quantitative profiling of genome-wide pre-mRNA AS (27). For instance, previous studies using RNA-Seq have reported that the infection of human macrophages with *Mycobacterium tuberculosis* results in massive alterations in the pattern of AS in human macrophages and in epithelial and macrophage cell lines (28, 29). In cattle, the differential AS patterns of transcript sequences between healthy cows and those naturally infected with *Staphylococcus aureus* were compared to understand the molecular mechanisms underlying mastitis resistance and susceptibility (30, 31). Using surgically isolated intestinal segments, significant changes in AS events between MAP-infected and non-infected tissues within the same calf were detected 1-month post-infection (32). In the present study, the absence or presence of PTB-associated lesions was used to define the stage of MAP infection and compare the AS profiles of animals with focal or diffuse lesions *vs.* uninfected cattle without lesions in gut tissues. Four of the identified AS events were experimentally validated by reverse transcription-digital droplet PCR (RT-ddPCR). Next, we assessed whether the AS events identified in the present study impacted gene expression. Subsequently, genes with changes in AS events and mRNA expression between animal groups were used in functional analysis to identify enriched gene ontologies and metabolic pathways in cows with lesions of distinct severity.

## 2 Materials and methods

### 2.1 Ethics statement

The study is reported in accordance with ARRIVE guidelines (https://arriveguidelines.org **).** The Animal Ethics Committee of the Servicio Regional de Investigation y Desarrollo Agroalimentario (SERIDA) approved the procedures on the animals included in this study. All procedures were authorized by the Regional Consejería de Agroganadería y Recursos Autóctonos of the Principality of Asturias (approval code PROAE 29/2015 and PROAE 66/2019) and were carried out following the European Guidelines for the Care and Use of Animals for Research Purposes (2012/63/EU). PB, gut tissues, and fecal samples were collected by trained personnel and in accordance with good veterinary practices.

### 2.2 Animals and PTB diagnosis

Peripheral blood (PB) and ileocecal valve (ICV) samples were collected from 14 female Holstein Friesian cows from a single commercial dairy farm in Asturias (Spain) at the time of slaughter. Their PTB infection status was determined by histopathological analysis of gut tissues. The Mycobacterium paratuberculosis Antibody test (IDEXX laboratories, Hoofddrop, the Netherlands) was used for the detection of MAP antibodies. Fecal and gut tissue bacteriological culture and PCR were carried out as previously described (13, 33). The post-mortem diagnostic results are displayed in **Supplementary Table 1**. All control animals (N= 4) showed a negative result for all diagnostic tests. In contrast, the infected group had either focal (N= 5) or diffuse (N= 5) lesions in gut tissues. The average age of the animals without lesions and with focal and diffuse lesions was 5.45, 5.09, and 4.38 years old, respectively.

### 2.3 RNA extraction, RNA-Seq library preparation, sequencing, and differential gene expression analysis

RNA extraction from PB and ICV samples, RNA-Seq library preparation, and sequencing were performed as previously described (13). Briefly, total RNA was purified from the PB samples using the PAXgene blood RNA kit according to the manufacturer´s instructions (Qiagen, Hilden, Germany). For RNA isolation, 150–200 mg of ICV was harvested and immediately submerged in 2 ml of RNAlater (Sigma-Aldrich, St. Louis, MO). Purification of RNA was performed using an RNeasy Mini Kit according to the manufacturer’s instructions (Qiagen, Hilden, Germany). Approximately 250 ng of RNA was used for RNA-Seq library preparation using an Illumina NEBNext Ultra Directional RNA Library preparation kit following the manufacturer´s instructions (Illumina, San Diego, CA, USA). RNA-Seq libraries were single-end sequenced in a 1 × 75 bp format using an Illumina NextSeq 500 sequencer at the Genomic Unit of the Madrid Science Park, Spain. Quality control of the sequence data was assessed using *FASTQC* 0.11.9 (https://www.bioinformatics.babraham.ac.uk/projects/fastqc/). The 13 bp Illumina standard adapters were removed using the trimming software *Trim Galore!* 0.6.5. (https://github.com/FelixKrueger/TrimGalore). Trimmed reads were subsequently aligned to the *Bos taurus* reference genome (ARS-UCD1.2, INSDC Assembly GCA_002263795.2) using the Spliced Transcripts Alignment to a Reference aligner (*STAR)* 2.5.3a software (34). In addition to the unbiased *de novo* detection of canonical junctions, *STAR* can discover alternative splices and chimeric transcripts and can map full-length RNA sequences. The program required a GTF file containing all *Bos taurus* coding transcripts, which was downloaded from Ensembl (Bos_taurus.ARS-UCD1.2.100.gtf). For each library, the resulting alignments (.bam files) were used to generate a table of counts for each gene using the *FeatureCounts* function from the *Rsubread* 2.4.2. package (35). Gene counts were then normalized with the mean-of-ratios method included in the *DESeq2* 1.30.0 package (36). *DESeq2* was also used to perform differential gene expression analysis for all comparisons: cows with focal lesions *vs.* controls, diffuse lesions *vs.* controls, and diffuse *vs.* focal lesions. A gene was considered as differentially expressed if its false discovery rate (FDR)-adjusted *P*-value (P_FDR_) was lower than 0.05 after correction for multiple testing using the Benjamini–Hochberg method (37).

### 2.4 Identification of differential AS events between infected and control cows

**Figure 2:**
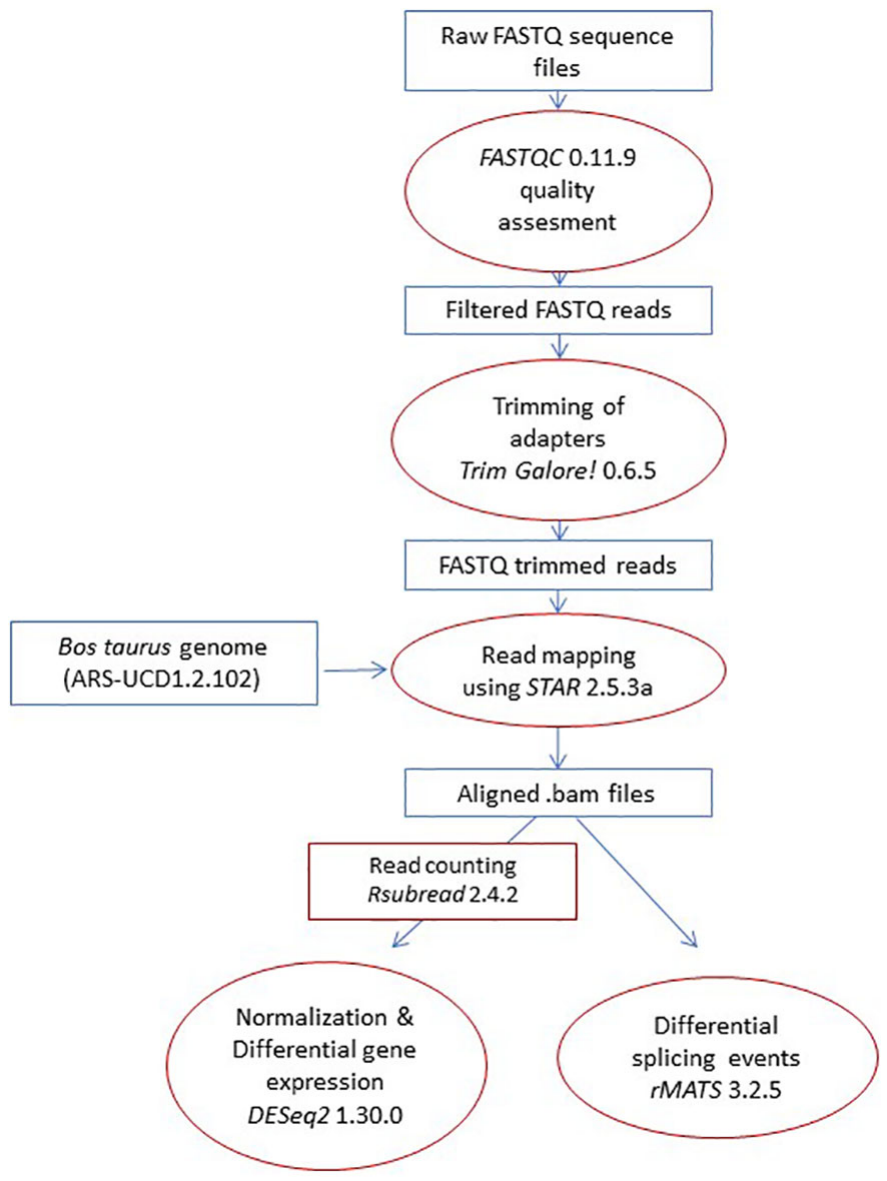
Pipeline. The pipeline started with the raw reads, which were mapped to the *Bos taurus* genome. Next, AS events between groups were compared. Finally, genes with differential AS and expression between groups were identified and used in functional analysis.

Using the mapped reads (.bam) and Ensembl’s annotation gtf file as inputs, the replicate multivariate analysis of transcript splicing software (*rMATS* 3.2.5) was used to detect differential AS events between RNA-Seq samples. *rMATS* annotates the AS types (SE, MXE, A3SS, A5SS, and RI), uses a hierarchical framework to account for estimation uncertainty in individual replicates, and models exon inclusion levels, referred to as percent spliced-in (PSI) (38). *rMATS* normalizes the lengths of individual splice variants and uses a likelihood-ratio test to calculate whether the difference in the mean PSI values between two sample groups is significant. The pipeline used for differential gene expression and AS analysis is described in **Figure 2**.

### 2.5 Validation of Identified Differential AS Events by reverse transcription-digital droplet PCR

The quantification of exon inclusion levels in four of the AS events identified with *rMATS* was carried out using RT-ddPCR. Two primer pairs were designed for the validation of each event; one to amplify the exon present in each event type, and the other to amplify the exon present in all isoforms annotated in the Ensembl database. First, 10 ng of total RNA was reverse transcribed into cDNA using an RT2 First Strand Kit according to the manufacturer´s instructions (Qiagen, Hilden, Germany). The reaction mixes of each ddPCR assay included 10 µl of 2x QX200™ ddPCR™ EvaGreen® Supermix (Bio-Rad, Hercules, CA, USA), 1µl of 4 µM of each primer, 1 µl of cDNA, and 7 µl of DEPC-treated H_2_O. A negative control without DNA was also included. Each mixed sample was pipetted into a DG8™ Cartridge (Bio-Rad, USA) previously loaded with 70 µl of droplet generation oil for Eva Green ® (Bio-Rad, USA). The cartridge was covered with a DG8™ Gasket (Bio-Rad, USA) and placed into a QX200 Droplet Generator (Bio-Rad, USA) to perform droplet generation. The droplets were transferred to a 96-well plate (Bio-Rad, USA) with a RAININ p-50 pipette, and the plate was sealed with pierceable foil at 180°C for 5 s using a PX1™ PCR Plate Sealer (Bio-Rad, USA). The PCR plate was placed in a T100™ Thermal Cycler (Bio-Rad, USA) for PCR using the following cycling conditions: an initial denaturation cycle at 95°C for 30 s was followed by 40 cycles of 30 s at 95°C and 1 min at 60°C. A final signal stabilization cycle at 4°C for 5 min followed by 90°C at 5 min was performed. The plate was then read using a QX200 Droplet Reader (Bio-Rad, USA), and the quantification of target DNA was performed using *QuantaSoft*™ *Analysis Pro* (version 1.0.596) (Bio-Rad, USA). The concentration of each DNA sample in the original reaction was obtained using the following formula:


$$\frac{\text{copies}}{\mu \text{l}}(\text{in original sample})=\frac{(\frac{\text{copies}}{\mu \text{l}}(\text{in ddPCR reaction}))\text{A}}{\text{B}}$$

A: *Total volume of the ddPCR reaction mix (µl)*

B: *Total DNA loaded to the ddPCR reaction mix (µl)*

The PSI value of each event was calculated by dividing the copies/µl obtained from the ddPCR of the inclusion isoform by the copies/µl obtained from the ddPCR of the sequence present in all isoforms. Finally, the ΔPSI for each comparison was calculated as the average of the PSIs of the control group minus the average of the PSIs of the samples from cows with focal or diffuse lesions. For the comparisons of cows with diffuse *vs.* focal lesions, the average of the PSIs of the focal group minus the average of the PSIs of the samples from cows with diffuse lesions was calculated.

### 2.6 Functional enrichment analysis

Genes with differential AS events and mRNA expression between groups were investigated for the enrichment of gene ontologies (biological processes, cellular components, and biological functions) and *KEGG* pathways using the *ClusterProfiler* package (39) and the *STRING* database (40). The Benjamini–Hochberg method was applied to adjust for multiple testing, considering a P_FDR_≤ 0.05 as significant. The function of the candidate genes was identified in *GeneCards* by searching for their gene symbol.

## 3 Results

### 3.1 AS events of pre-mRNA in PB and ICV from MAP-infected animals *vs.* controls

**Figure 3:**
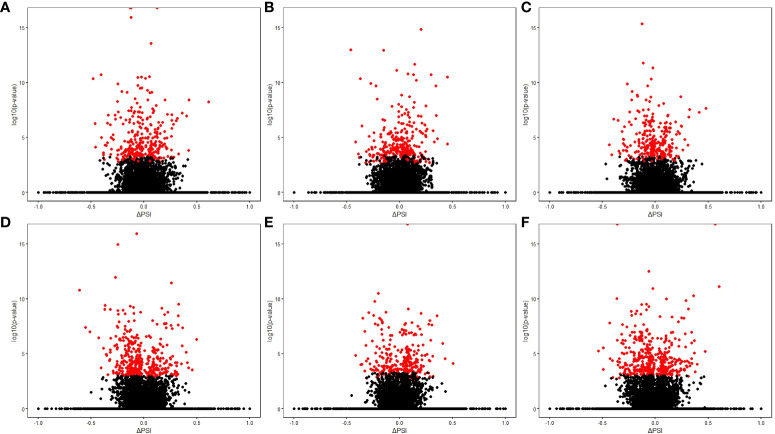
Results of the AS differential analysis. Volcano plots representing the differential AS events (∆PSI value *vs* –log_10_ [p-value]) for each comparison. **(A)** PB samples of cows with focal lesions *vs.* controls. **(B)** PB samples of cows with diffuse lesions *vs.* controls. **(C)** PB samples of cows with diffuse *vs.* focal lesions. **(D)** ICV samples of cows with focal lesions *vs.* controls. **(E)** ICV samples of cows with diffuse lesions *vs.* controls. **(F)** ICV samples of cows with diffuse *vs.* focal lesions. The red spots represent the differential AS events in each comparison (P_FDR_≤ 0.05).

**Figure 4:**
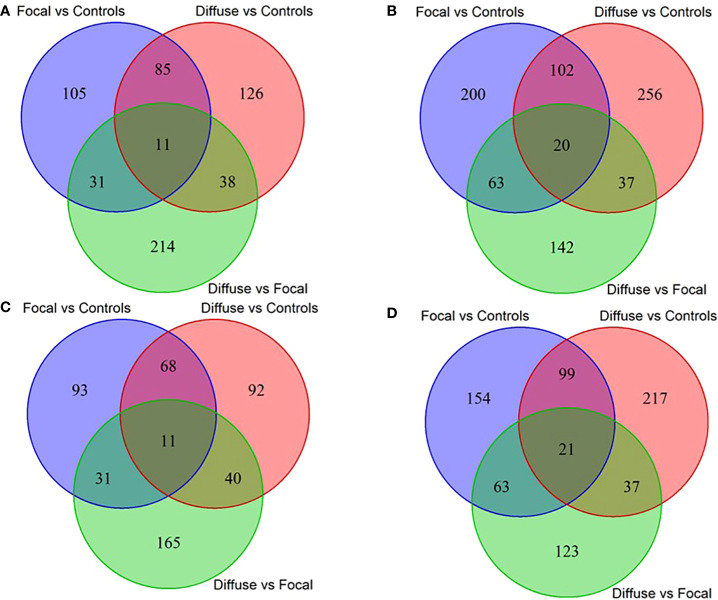
Venn diagrams showing the number of differential and common AS events and target genes between the three comparisons. Number of differential and common AS events in PB **(A)** and ICV **(B)** samples from cows with focal lesions *vs*. controls, with diffuse lesions *vs.* controls, and with diffuse *vs.* focal lesions. Number of differential and common AS genes in PB **(C)** and ICV **(D)** samples from cows with focal lesions *vs*. controls, with diffuse lesions *vs* controls, and with diffuse lesions *vs.* focal lesions.

Using RNA-Seq data from the animals included in the study, AS differences in PB and ICV samples between infected cows with focal or diffuse lesions *vs.* controls and between cows with diffuse *vs.* focal lesions were identified using the software *rMATS* 3.2.5 (38). Using the *rMATS* output, we annotated the number and frequency of each AS event (A5SS, A3SS, RI, MXE, and SE) by calculating the PSI value, which represents the frequency in which a specific exon is included in the final transcript. The cut-off for the detection of significant AS events was set at P_FDR_≤ 0.05. Our results evidenced differential AS patterns between infected animals with focal and diffuse PTB-associated lesions *vs.* the control animals and with diffuse *vs.* focal lesions (**Figure 3**). We found a higher number of AS events in ICV samples than in PB samples. In the PB samples, 232, 260, and 294 AS events were identified in the comparisons of cows with focal lesions *vs.* controls, diffuse lesions *vs.* controls, and diffuse *vs.* focal lesions, respectively (**Figure 4A**). AS profiling of ICV samples showed 385, 415, and 262 AS events in the comparison of cows with focal lesions *vs.* controls, diffuse lesions *vs.* controls, and diffuse *vs.* focal lesions, respectively (**Figure 4B**). The number of alternatively spliced genes identified in PB and ICV samples for each comparison are presented in **Figures 4C, D**, respectively. The total number of significant AS events found in all comparisons is presented in **Table 1**, the most frequent type being SE, followed by MXE and RI, and the less frequent ones being A3SS and A5SS. Data analysis revealed 96 and 122 AS events that were common in the PB and ICV samples, respectively, of the infected animals regardless of the type of lesion. A total of 26 and 38 AS events were common in PB and ICV samples of the animals with focal and diffuse lesions *vs.* control cows, respectively. Finally, 16 AS events were common in the PB and ICV samples of the infected animals regardless of the specific type of lesion. Although some AS events were common in PB and ICV samples, we also found tissue-specific AS events. **Table 2** shows the five AS events with the highest ΔPSI for each comparison.

**Table 1:** Differential AS events in each comparison and common AS events between comparisons.

Sample	Comparison	AS type					Number of AS events	Number of AS genes
		A3SS	A5SS	MXE	RI	SE		
PB	Focal *vs.* controls	4	5	25	16	182	232	203
PB	Diffuse *vs.* controls	6	5	49	19	181	260	211
PB	Diffuse *vs.* focal	7	1	123	18	145	294	247
PB	Focal *vs.* controls + diffuse *vs.* controls	1	2	12	4	77	96	79
ICV	Focal *vs.* controls	6	1	53	24	301	385	337
ICV	Diffuse *vs.* controls	4	5	67	26	313	415	374
ICV	Diffuse *vs.* focal	7	4	52	42	157	262	244
ICV	Focal *vs.* controls + diffuse *vs.* controls	0	1	18	0	103	122	120
PB+ICV	Focal *vs.* controls	0	0	5	1	20	26	26
PB+ICV	Diffuse *vs.* controls	0	1	7	0	30	38	35
PB+ICV	Diffuse *vs.* focal	1	0	3	2	12	18	18
PB+ICV	Focal *vs.* controls + diffuse *vs.* controls	0	0	4	0	12	16	16

PB, peripheral blood; ICV, ileocecal valve; A3SS, alternative 3′ splice site; A5SS, alternative 5′ splice site; MXE, mutually exclusive exons; RI, retained intron; SE, skipped exon. “and” is represented as (+).

**Table 2:** The five AS events with the highest ΔPSI in PB and ICV samples from MAP-infected cows *vs.* controls and between cows with diffuse lesions *vs.* focal lesions.

Sample	Comparison	Event ID	Event type	Gene ID	Gene Symbol	ΔPSI	P_FDR_
PB	Focal lesions *vs.* controls	ev17006	SE	ENSBTAG00000007097	THTPA	0.441	2.99E-02
		ev16794	SE	ENSBTAG00000015376	FBXW11	0.382	4.74E-04
		ev920	SE	ENSBTAG00000021761	BRAF	0.376	2.03E-03
		ev188	SE	ENSBTAG00000012126	PNPLA7	0.346	4.96E-03
		ev14617	SE	ENSBTAG00000016900	TRAPPC13	0.321	1.14E-05
PB	Diffuse lesions *vs.* controls	ev7202	SE	ENSBTAG00000020595	KCTD20	0.479	2.80E-05
		ev2515	MXE	ENSBTAG00000004815		0.414	2.03E-05
		ev5442	SE	ENSBTAG00000039702	FCRL3	0.323	3.23E-05
		ev6205	SE	ENSBTAG00000015007	NCOA1	0.316	9.26E-05
		ev1407	MXE	ENSBTAG00000019458	ASB3	0.307	4.25E-03
PB	Diffuse *vs.* focal lesions	ev8174	SE	ENSBTAG00000013314	PBX3	0.452	6.51E-08
		ev2591	MXE	ENSBTAG00000000149	USP40	0.451	3.85E-03
		ev1644	MXE	ENSBTAG00000014714	TUBGCP5	0.359	1.87E-03
		ev4534	SE	ENSBTAG00000014680	ZBTB49	0.347	7.21E-05
		ev1704	MXE	ENSBTAG00000002069	BOLA	0.343	5.10E-07
ICV	Focal lesions *vs.* controls	ev679	SE	ENSBTAG00000012552	FMR1	0.496	1.36E-04
		ev3168	SE	ENSBTAG00000004564	MBNL1	0.455	1.84E-02
		ev1597	MXE	ENSBTAG00000014178	RALGAPA2	0.421	1.38E-02
		ev9254	SE	ENSBTAG00000020653	FIP1L1	0.412	5.97E-03
		ev1037	SE	ENSBTAG00000000224	TSPAN15	0.400	1.06E-03
ICV	Diffuse lesions *vs.* controls	ev6709	SE	ENSBTAG00000014546	CLEC7A	0.605	3.27E-08
		ev8773	SE	ENSBTAG00000039995		0.567	0
		ev15724	SE	ENSBTAG00000004461	OFD1	0.472	1.15E-03
		ev6246	SE	ENSBTAG00000002130	SMPD4	0.396	8.64E-03
		ev873	MXE	ENSBTAG00000007545	PHLPP2	0.377	2.38E-02
ICV	Diffuse *vs.* focal lesions	ev765	RI	ENSBTAG00000012113	HCCS	0.504	2.54E-03
		ev12906	SE	ENSBTAG00000006025	AHSA2	0.429	6.01E-03
		ev784	RI	ENSBTAG00000006974	PLEKHA7	0.408	1.22E-04
		ev18077	SE	ENSBTAG00000018316	ZMYND15	0.355	8.00E-06
		ev533	MXE	ENSBTAG00000016553	EME2	0.316	4.43E-02

PB, peripheral blood; ICV, ileocecal valve; A3SS, alternative 3′ splice site; A5SS, alternative 5′ splice site; MXE, mutually exclusive exons; RI, retained intron; SE, skipped exon; P_FDR_, false discovery rate (FDR)-adjusted P-value. ΔPSI for each comparison was calculated as the average of the PSIs (percent spliced-in) of the control group minus the average of the PSIs of the target group.

### 3.2 Validation of some of the identified AS events in PB samples by RT-ddPCR

**Figure 5:**
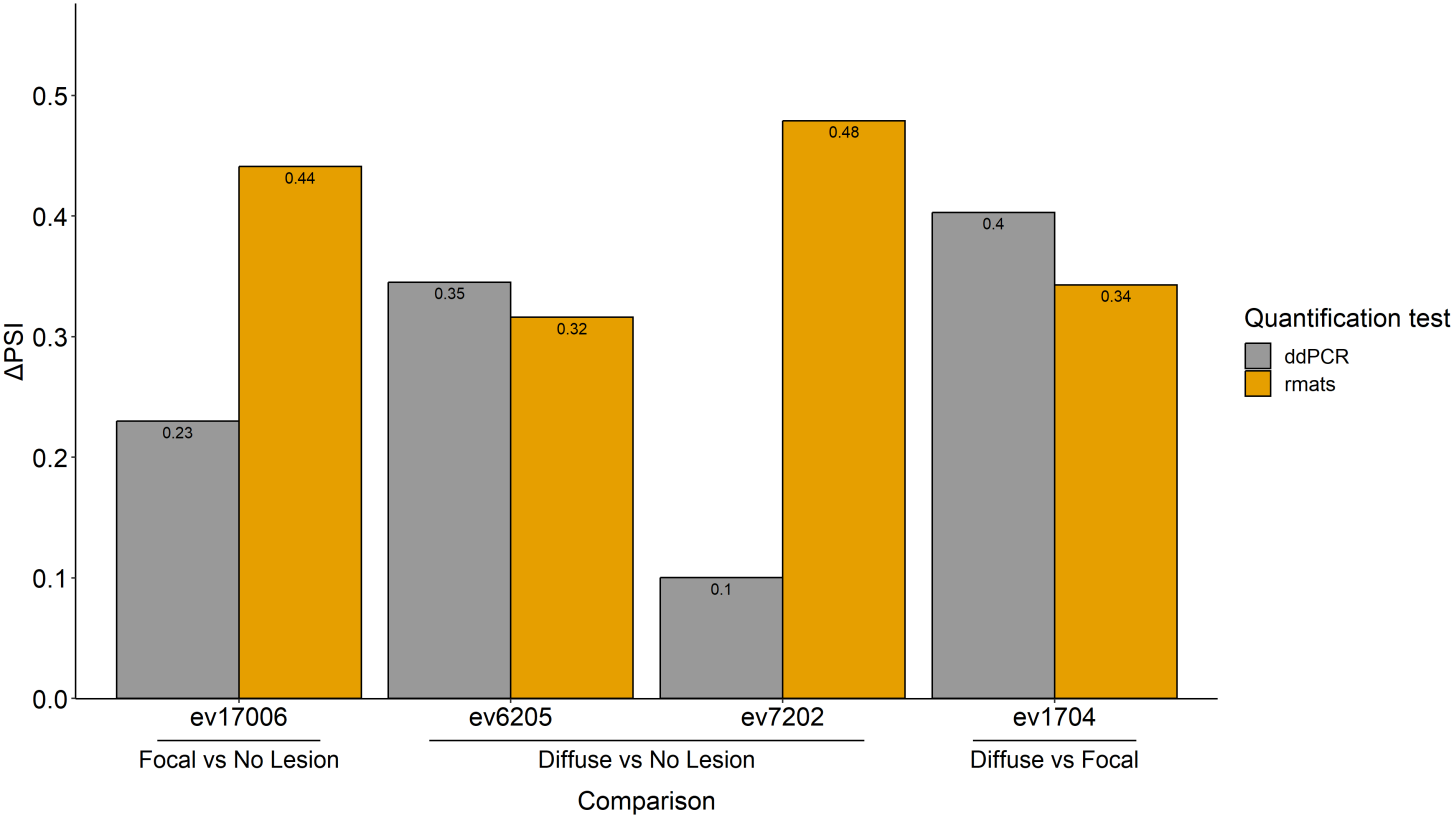
RT-ddPCR validation of four AS events identified *in silico* with *rMATS*. Four of the AS events with the highest ΔPSIs, ev17006, ev6205, ev7202, and ev1704, were experimentally validated by RT-ddPCR. Two primer pairs were designed for the validation of each event; one to amplify the exon present in each event type, and the other to amplify the exon present in all isoforms annotated in the Ensembl database. The PSI value of each event was calculated by dividing the copies/µl obtained from the ddPCR of the inclusion isoform by the copies/µl obtained from the ddPCR of the sequence present in all isoforms. The ΔPSI for each comparison was calculated as the average of the PSIs of the control group minus the average of the PSIs of the samples from cows with focal or diffuse lesions. For the comparisons of cows with diffuse lesions *vs.* focal lesions, the average of the PSIs of the focal group minus the average of the PSIs of the samples from cows with diffuse lesions was calculated. Changes in the AS events (expressed as ΔPSI) calculated using RNA-Seq and RT-ddPCR are presented in yellow and grey, respectively.

AS events can potentially be used as disease biomarkers and their detection by RT-ddPCR can be performed in blood samples of live animals. Therefore, for further validation by RT-ddPCR, we selected four events among the AS events with the highest ΔPSI in PB samples. One of the selected events, ev17006 (SE-type, ΔPSI= 0.441), targeted the *Thiamine Triphosphatase* (*THTPA*) gene and was identified in the comparison of cows with focal lesions *vs.* controls. The ΔPSI of this SE event was positive, which means that the exon is more frequently skipped in cows with focal lesions than in the controls. Events ev7202 (ΔPSI= 0.479) and ev6205 (ΔPSI= 0.316) were identified in the comparison of cows with diffuse lesions *vs.* controls and targeted *Potassium Channel Tetramerization Domain Containing* (*KCTD20*) and *Nuclear Receptor Coactivator 1* (*NCOA1*), respectively. Both were SE events, had a positive ΔPSI, and, therefore, the exons were more frequently skipped in cows with diffuse lesions than in the controls. An MXE event (ev1704) with a positive ΔPSI (ΔPSI= 0.343) was identified in cows with diffuse *vs.* focal lesions, and it was selected for validation because this specific event targeted the *bovine leukocyte antigen* (*BOLA*) gene, which plays an important role in antigen processing and presentation. Two primer pairs were designed for the validation of each event (**Supplementary Table 2**); one to amplify the exon present in each event type, and the other to amplify the exon present in all isoforms annotated in the Ensembl database. The results of the RT-ddPCR assays are presented in **Figure 5** and were expressed as ΔPSI to be comparable with the *in silico* results obtained with *rMATS*. All the events analyzed with RT-ddPCR showed positive ΔPSI values, as previously obtained with *rMATS*.

### 3.3 Functional analysis of the alternatively spliced genes

We performed a functional analysis with the genes undergoing AS for each comparison using *ClusterProfiler* and *STRING*. The enriched GOs and pathways identified in the PB and ICV samples are presented in **Supplementary Tables 3** and **4**, respectively. In the PB samples, the AS events identified in cows with focal lesions *vs.* controls affected genes playing important roles in endocytosis (bta:04144) and associated with viral infections caused by viruses able to establish long latent infections, such as the Epstein–Barr virus (EBV) (bta:05169) and Kaposi sarcoma-associated herpesvirus infection (bta:05167). These three pathways were enriched in three common alternatively spliced genes; *BOLA*, class I histocompatibility antigen Gogo-C*0202 alpha chain (LOC509006), and *JNK-stimulatory Phosphatase-1* (JSP.1). In the comparison of cows with diffuse lesions *vs.* controls, we detected the enrichment of several genes with AS events, such as *leukocyte surface Antigen CD53*, *Dendritic Cell-Associated Lectin 2* (*CLEC12A*), *Natural Resistance-Associated Macrophage Protein 1* (*SLC11A1*), and *T Cell-Interacting, Activating Receptor on Myeloid Cells 1* (*TARM1*) involved in clathrin (CLTA)-mediated endocytosis, neutrophil degranulation, and platelet activation. In the comparison of cows with diffuse *vs.* focal lesions, enrichment of AS events in genes belonging to the lysosome (bta:04142) and amoebiasis (bta:05146) pathways was detected. In the ICV samples of the infected cows, genes that encode for proteins with RNA-binding and coiled-coil domains showed differential AS patterns when compared with the control group. In the comparison of cows with diffuse *vs.* focal lesions, enrichment of genes that encode for proteins with coiled-coil domains and bromodomains and involved in AS was found.

### 3.4 Identification of genes displaying changes in AS and gene expression

**Figure 6:**
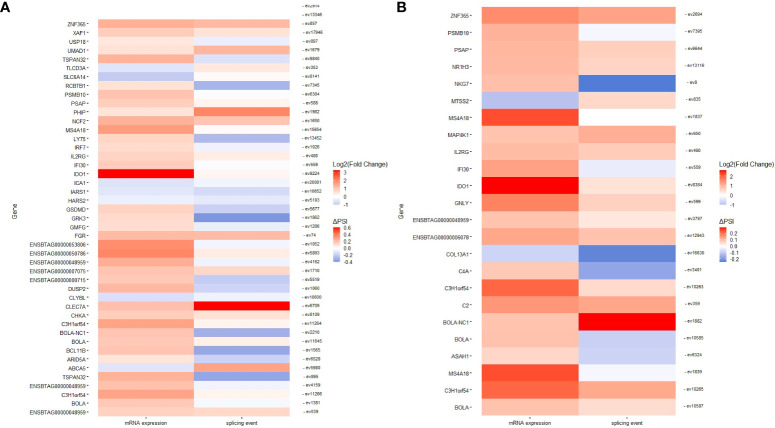
Genes displaying changes in AS and gene expression. Heatmaps representing genes with changes in AS and expression in ICV samples of cows with diffuse lesions *vs.* controls **(A)** and diffuse lesions *vs.* focal lesions **(B)**.

In the PB samples of cows with focal lesions *vs.* controls, we did not identify genes displaying changes in AS and gene expression. The genes displaying changes in AS and gene expression are shown in **Supplementary Table 5**. In the PB samples from cows with diffuse lesions *vs.* controls, we found two SE events (ev75562 and ev14014) with negative ΔPSI values (less frequent in cows with diffuse lesions than in the controls) that were associated with the downregulation of *Flotillin-1* (*FLOT1*) (fold change= -1.186), a gene involved in the formation of caveolae-like vesicles, and *Triggering Receptor Expressed On Myeloid Cells Like 2* (*TREML2*) (fold change= -1.081), a counter-receptor for CD276 that enhances T-cell activation, respectively. The event ev3194 was less frequent in cows with diffuse lesions than in controls (ΔPSI= −0.26) and associated with the dysregulation of *Cytochrome P450 Family 4 Subfamily V Member 2 (CYP4V2)* (fold change= 0.071), a gene involved in fatty acid metabolism. In the comparison of cows with diffuse *vs.* focal lesions, ev13968 was most frequent in cows with diffuse lesions (ΔPSI= 0.004) and associated with the downregulation of *Pepsinogen A5* (*PGA5*) (fold= −2.406), which functions in the digestion of dietary proteins. In the ICV samples, 1, 47, and 24 events had a differential frequency of appearance in the comparisons of cows with focal lesions *vs.* controls, diffuse lesions *vs.* controls, and diffuse *vs.* focal lesions, respectively. In the first of these comparisons, the SE ev1886 (ΔPSI= −0.277) was less frequent in cows with focal lesions than in the controls and was associated with the dysregulation of *Cytochrome P450 Family 4 Subfamily F Member 2* (*CYP4F2*) (fold= 1.097), an enzyme that starts the process of inactivating and degrading leukotriene B4, a potent mediator of inflammation. The AS events identified in the comparisons of cows with diffuse lesions *vs.* controls and diffuse *vs.* focal lesions and the effect of these events on gene expression are graphically presented in **Figures 6A, B**, respectively. As seen in **Figure 6**, most of the AS events were associated with the upregulation of the expression of their target genes. Some genes involved in antigen-presentation and processing and inflammatory response, including *BOLA*, *BOLA-NC1*, *Interferon Gamma-Inducible Protein 30 (IFI30)*, *Interleukin 2 Receptor Subunit Gamma (IL2RG)*, and *Indoleamine 2,3-Dioxygenase 1 (IDO1)*, were upregulated and affected by changes in AS in both comparisons.

### 3.5 Functional analysis of genes displaying changes in both AS and gene expression

We performed a functional analysis with the genes displaying changes in both AS and gene expression. As seen in **Table 3**, GOs and metabolic pathways were only identified in the ICV samples from cows with diffuse lesions *vs.* controls and with diffuse *vs.* focal lesions. In both comparisons, truncated transcripts correlated with the increased expression levels of several genes, including *BOLA* and *BOLA-NC1*, which are involved in endocytosis (bta:04144), antigen processing and presentation (bta:04612), type I diabetes mellitus (bta04940), allograft rejection (bta05330), graft-versus-host disease (bta:05332), autoimmune thyroid disease (bta:05320), viral myocarditis (bta05416), and human T cell leukemia virus 1 infection (bta:05166). In the comparisons of cows with diffuse *vs.* focal lesions, AS events were associated with the upregulation of the complement *C2* and *C4A* genes involved in complement activation (GO:006956 and GO:006958), humoral immune response mediated by circulating immunoglobulins (Ig) (GO:0002455), Igs-mediated immune response (GO:00160664), and B-cell-mediated immunity (GO:0019724), and with the pathogenesis of several human diseases such as Pertussis (*bta:05133*), an acute respiratory infectious disease caused by *Bordetella Pertussis*, and *Staphylococcus aureus* infection (bta:05150). In the comparison of cows with diffuse *vs.* focal lesions, three genes (*IFI30*, *PSAP*, and *ASAH1*) involved in the lysis of vacuoles by lysosomes and MHC class II-restricted antigen processing showed changes in AS and were upregulated in cows with diffuse lesions *vs.* focal lesions.

**Table 3:** Functional analysis using the genes with AS and mRNA expression dysregulation in the ICV samples.

Comparison	ID	Description	P_FDR_	Gene ID
Diffuse lesions *vs.* controls	bta04940	Type I diabetes mellitus	0.001	ICA1/BOLA/LOC616942/BOLA-NC1
	bta04145	Phagosome	0.002	BOLA/LOC616942/NCF2/CLEC7A/BOLA-NC1
	bta04612	Antigen processing and presentation	0.002	BOLA/LOC616942/IFI30/BOLA-NC1
	bta04144	Endocytosis	0.006	GRK3/BOLA/LOC616942/IL2RG/BOLA-NC1
	bta05330	Allograft rejection	0.006	BOLA/LOC616942/BOLA-NC1
	bta05332	Graft-versus-host disease	0.007	BOLA/LOC616942/BOLA-NC1
	bta05320	Autoimmune thyroid disease	0.008	BOLA/LOC616942/BOLA-NC1
	bta05416	Viral myocarditis	0.008	BOLA/LOC616942/BOLA-NC1
	bta05167	Kaposi sarcoma-associated herpesvirus	0.017	BOLA/LOC616942/BOLA-NC1/IRF7
	bta05166	Human T-cell leukemia virus 1	0.018	BOLA/LOC616942/IL2RG/BOLA-NC1
	bta05169	Epstein–Barr virus	0.018	BOLA/LOC616942/BOLA-NC1/IRF7
	bta05170	Human immunodeficiency virus 1	0.018	BOLA/LOC616942/BOLA-NC1/LOC618737
	bta05203	Viral carcinogenesis	0.018	BOLA/LOC616942/BOLA-NC1/IRF7
	bta05168	Herpes simplex virus 1	0.018	BOLA/LOC616942/BOLANC1/IRF7/LOC618737
	bta04514	Cell adhesion molecules	0.035	BOLA/LOC616942/BOLA-NC1
	bta04218	Cellular senescence	0.035	BOLA/LOC616942/BOLA-NC1
	bta00970	Aminoacyl-tRNA biosynthesis	0.047	IARS1/HARS2
Diffuse *vs.* focal lesions	GO:0006958	Complement activation, classical pathway	0.034	C2/C4A
	GO:0002455	Humoral immune response mediated by Ig	0.034	C2/C4A
	GO:0006956	Complement activation	0.034	C2/C4A
	GO:0002376	Immune system process	0.034	IFI30/PSMB10/NR1H3/C2/C4A
	GO:0006665	Sphingolipid metabolic process	0.034	PSAP/ASAH1
	GO:0016064	IgG-mediated immune response	0.034	C2/C4A
	GO:0019724	B-cell-mediated immunity	0.034	C2/C4A
	GO:0000323	Lytic vacuole	0.045	IFI30/PSAP/ASAH1
	GO:0005764	Lysosome	0.045	IFI30/PSAP/ASAH1
	bta04612	Antigen processing and presentation	0.009	IFI30/BOLA/BOLA-NC1
	bta05166	Human T-cell leukemia virus 1 infection	0.009	IL15RA/BOLA/IL2RG/BOLA-NC1
	bta00600	Sphingolipid metabolism	0.030	PSAP/ASAH1
	bta05330	Allograft rejection	0.030	BOLA/BOLA-NC1
	bta04940	Type I diabetes mellitus	0.030	BOLA/BOLA-NC1
	bta05332	Graft-versus-host disease	0.030	BOLA/BOLA-NC1
	bta05320	Autoimmune thyroid disease	0.030	BOLA/BOLA-NC1
	bta04144	Endocytosis	0.030	BOLA/IL2RG/BOLA-NC1
	bta05133	Pertussis	0.030	C2/C4A
	bta05416	Viral myocarditis	0.030	BOLA/BOLA-NC1
	bta04610	Complement and coagulation cascades	0.038	C2/C4A
	bta05150	Staphylococcus aureus infection	0.046	C2/C4A

## 4 Discussion

Recent advances in high-throughput sequencing RNA technology and computational tools have greatly facilitated AS profiling (41). Using RNA-Seq data from the peripheral blood, jejunum, and salivary gland of Holstein cows classified as positive or negative according to ELISA and fecal PCR results, 119, 150, and 68 differential AS events were identified, respectively (32). In this study, however, only 14 alternatively spliced genes were significantly enriched for immune-related pathways. PTB-associated focal lesions in gut tissues can be detected before fecal shedding and MAP antibodies, and therefore, examining the AS profiles of animals according to histopathological results facilitates the identification of animals in the subclinical stage of the infection when the lesions in gut tissues remain focalized and the amount of MAP and anti-MAP antibodies are undetected with current pre-mortem diagnostic methods. To the best of our knowledge, our study is the first genome-wide profiling of AS events in PB and ICV from Holstein cows with PTB-associated lesions of distinct severity *vs.* uninfected cattle without lesions in gut tissues.

**Figure 7:**
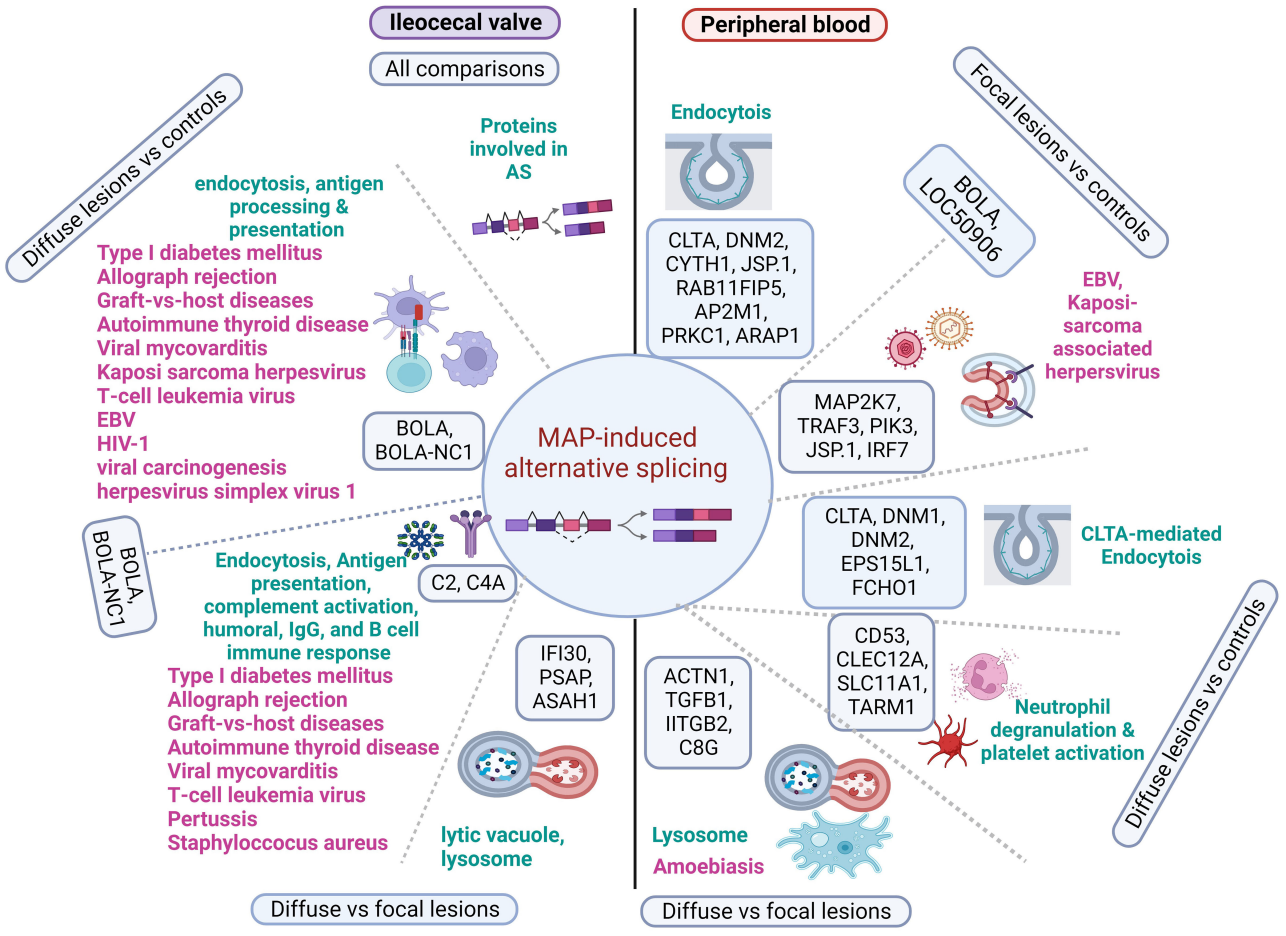
Diagram showing the main alternatively spliced genes involved in MAP immunopathogenesis and several human diseases. Alternatively spliced genes and pathways identified in the peripheral blood and ileocecal valve of cattle with distinct PTB-associate lesions are represented in the right and left side of the figure, respectively. In the PB of cows with focal lesions, alternatively spliced genes involved in the endocytosis and recognition of antigens (*BOLA* and *LOC50906*) might contribute to immune tolerance to MAP antigens by similar mechanisms, as observed in EBV and Kaposi sarcoma-associated herpesvirus infections. Although the AS of genes involved in neutrophil degranulation and platelet activation may control MAP growth and stimulate the development of granulomas, AS of genes involved in the lysis of MAP-containing phagosomes might lead to apoptosis and disruption of the intestinal mucus layer by similar mechanisms as seen in humans infected with *Entamoeba histolytica*. In ICV samples of cattle with diffuse lesions, AS and the upregulation of genes involved in endocytosis and antigen processing and presentation (*BOLA* and *BOLA-NC1*) might activate an uncontrolled pro-inflammatory response. Uncontrolled antigen presentation and processing and complement activation have been associated with several human inflammatory and autoimmune human diseases. Alternatively spliced genes, enriched pathways, and associated human diseases are present in black, green, and pink, respectively. Created with BioRender.com.

Although MAP infection has been associated with several inflammatory and autoimmune diseases in humans, common mechanisms linking the pathogenesis of these diseases have not been proposed. AS is a highly controlled cellular process and most disruptions in AS events lead to autoimmunity and pathological inflammatory conditions (26, 42). In our study, we discovered that many of the identified alternatively spliced genes belonged to families or functional groups related to the host immune response. This suggests that some of the identified AS events might be coordinately regulated to achieve more robust control over the activity of the immune system. Interestingly, we observed that overlapping mechanisms affected by AS were associated with the pathogenesis of PTB and several infectious, inflammatory, and autoimmune diseases in humans. In **Figure 7**, we present a graphical summary of the identified alternative spliced genes and key molecular mechanisms at the crosstalk of PTB and human inflammatory and autoimmune diseases. As it has been demonstrated that AS plays a major role in many human diseases through the manipulation of essential/functional protein domains with bovine orthologues, MAP-infected cattle could be used as a useful model for these human diseases (43).

In the comparison of PB samples from cows with focal lesions *vs.* controls, several of the AS events identified in this comparison targeted genes involved in different endocytosis (bta:04144) steps, including *BOLA*, LOC509006, *clathrin* (*CLTA)*, *Dynamin-2* (*DNM2*), *CLTA Adaptor Complex AP2, Mu Subunit* (*AP2M1*), *Cytohesin 1 (CYTH1*), *Dual Specificity Phosphatase 22* (JSP.1), *RAB11 Family Interacting Protein 5* (*RAB11FIP5*), the apoptosis regulator (*PRKC1*), *ArfGAP With RhoGAP Domain*, and *Ankyrin Repeat And PH Domain 1* (*ARAP1*). CLTA functions as the main structural component of the lattice-type cytoplasmic face of coated pits and vesicles that entrap specific macromolecules during receptor-mediated endocytosis. DNM2 is responsible for the release of CLTA-coated vesicles from the plasma membrane and AP2M1 is required for the activity of a vacuolar ATPase, which is responsible for proton pumping that occurs in the acidification of endosomes and lysosomes. CYTH1 regulates the adhesiveness of integrins at the plasma membrane and is involved in membrane trafficking and PRKC1 negatively regulates apoptosis. After entry into the host cell by endocytosis, MAP can survive in infected macrophages within phagosomes by inhibiting apoptosis and phagosome acidification, and by preventing the presentation of antigens to the immune system (6). In this context, our findings suggest that the AS of genes involved in the endocytosis pathway might contribute to phagosome maturation arrest and MAP persistence within the macrophages of cows with focal lesions in gut tissues. Similarly, infection by *Mycobacterium tuberculosis* (H37Rv strain) induced AS of RAB8B, a protein involved in intracellular vesicle transport, which helps the survival of H37Rv in THP1-infected macrophages (28).

In the comparison of PB samples from cows with focal lesions *vs.* controls, we also identified AS events in genes associated with the pathogenesis of viral infections caused by herpesvirus that infect human populations predominantly at an early age but remain mostly asymptomatic, such as the Kaposi sarcoma-associated herpesvirus infection (bta:05167) and EBV infection (bta:05169). Both viruses as MAP can establish a lifelong latency and survive against the host´s innate and adaptive immune mechanisms. More specifically, MAP infection results in the AS of some genes associated with the pathogenesis of Kaposi sarcoma-associated herpesvirus and EBV infections, including *BOLA*, *LOC509006*, *Mitogen-Activated Protein Kinase Kinase 7 (MAP2K7)*, *TNF Receptor Associated Factor 3* (*TRAF3*), *Interferon Regulatory Factor 7 (IRF7*), *Phosphatidylinositol-4,5-Bisphosphate 3-Kinase Catalytic Subunit Delta (PIK3CD)*, and *JSP*.1. IRF7 has been shown to play an important role in the regulation of EBV latency and EBV-encoded latent infection membrane protein-1 (LMP1) can interact with TRAF3 and several other members of the TRAF family (44). TRAF3 acts as a negative NF-κβ regulator, possibly to avoid an unregulated inflammatory response (45). Our findings suggest that the AS of the *BOLA*, *LOC509006*, *MAP2K7*, *TRAF3*, *IRF7*, *PIK3CD*, and *JSP.*1 genes might contribute to the development of focalized PTB-associated lesions and establishment of the latent stage of MAP infection as it has been observed in EBV and Kaposi sarcoma-associated herpesvirus latency. Interestingly, MAP and EBV were proposed as the main pathogens involved in the dysregulation pathways linked to RA (46).

In the comparison of PB samples from cows with diffuse lesions *vs.* controls, we identified AS genes involved in CLTA-mediated endocytosis, neutrophil degranulation, and platelet activation pathways. More specifically, AS of genes belonging to the neutrophil degranulation and platelet activation pathways, including *CD53*, *CLEC12A*, *SLC11A1*, and *TARM1*, was observed. CD53 contributes to the transduction of CD2-generated signals in T cells and natural killer cells and TARM1 increases the Toll-like receptor-mediated production of pro-inflammatory cytokines by macrophages and neutrophils. CLE12A and SLC11A1 (also known as *natural-resistance-associated macrophage protein 1* [*NRAMP1*]) are both associated with innate host resistance. SLC11A1 is a membrane transporter involved in iron metabolism that is expressed in late endosomal/lysosomal compartments of macrophages. Polymorphisms in this gene have been associated with susceptibility to mycobacterial diseases such as PTB, tuberculosis, and leprosy, human inflammatory diseases such as Crohn´s disease, and autoimmune diseases such as RA, MS, and T1DM (10). Kissler et al. demonstrated that *SLC11A1* gene silencing using RNAi in mice reduced the frequency of T1DM and protected against experimental autoimmune encephalomyelitis, demonstrating a role for *SLC11A1* in autoimmunity (47). In the PB of cows with diffuse lesions, AS events in antibacterial genes (*CD53*, *CLEC12A*, *SLC11A1*, and *TARM1)* involved in neutrophil degranulation and platelet activation were observed. Several *in vivo* studies demonstrated that neutrophils phagocytose Mycobacteria from dying Mycobacteria-infected macrophages within the nascent granuloma but simultaneously potentiate inflammation and tissue damage (48, 49). Similarly, uncontrolled activation of neutrophils has been shown to play an important role in human autoimmune and inflammatory diseases by exacerbating inflammation and tissue destruction (46). Therefore, the negative regulation of polymorphonuclear cell functions is anticipated to have significant pharmacological utility in treating the inflammation that is characteristic of the early clinical stage of PTB, as well as several human inflammatory and autoimmune illnesses (50).

In the comparison of PB from cows with diffuse lesions *vs.* focal lesions, we found several genes (*ACTN1, TGFβ2, ITGβ2*, and *C8G*) with AS events associated with the lysosome (bta:04142) and amoebiasis (bta:05146) caused by *Entamoeba histolytica*, a human extracellular protozoan parasite that like MAP invades the intestinal epithelium. The infection with *Entamoeba histolytica* occurs upon ingestion of contaminated water and food and involves parasite attachment and disruption of the intestinal mucus layer, followed by apoptosis of host epithelial cells (51). Intestinal tissue destruction causes severe dysentery and ulcerations in amoebic colitis. Moreover, the parasite can cause extraintestinal infections, such as amoebic liver abscesses, by evading the host immune response (52). MAP infection also impacts the intestinal mucosa and over time causes serious damage to the ileum and jejunum, diarrhea, progressive wasting, and the eventual death of the infected animal in the more advanced stages of clinical PTB (8). We propose that the AS of several genes associated with the activation of the lysosomes and amoebiasis might also cause disruption of the intestinal mucus layer, apoptosis, and MAP dissemination to other organs and tissues as seen in humans infected with *Entamoeba histolytica.* In the ICV samples of the infected cows, alternatively spliced genes that encode proteins with RNA-binding domains and coiled-coil domains critical for splicing activity, and involved in AS, were identified. As RNA splicing is regulated by cis-regulatory elements in pre-mRNA and trans-regulatory elements, mainly RNA-binding proteins, AS of RNA-binding proteins may in turn affect the splicing of other pre-mRNAs and contribute to the various onsets of PTB.

Our study also provides evidence of a relationship between AS patterns and gene expression regulation in distinct stages of MAP infection, as several genes were altered in both splicing and expression patterns in the ICV samples. In the ICV of animals with diffuse lesions, for instance, truncated transcripts correlated with increased expression levels of several genes involved in endocytosis (bta:04144) and antigen processing and presentation (bta:04612), including *BOLA* and *BOLA-NC1*. In cattle, the MHC region is termed *BOLA* and is involved in the processing and presentation of antigens to intestinal epithelial gamma delta T cells. *BOLA* genes are highly polymorphic, which leads to variation in the animal’s ability to recognize and present antigens, making some animals more susceptible to infection and disease than others. The human homologs of these genes have been also associated with several viral and autoimmune diseases, such as type I diabetes mellitus (bta:04940), Graft-versus-host disease (bta:05332), autoimmune thyroid disease (bta:05320), viral myocarditis (bta:05416), and human T cell leukemia virus 1 infection (bta:05166). In the comparisons of cows with diffuse lesions *vs.* focal lesions, AS events were associated with the upregulation of the *BOLA*, *BOLA-NC1*, and complement *C2* and *C4A* genes involved in complement activation (GO:006958 and GO:006958), humoral immune response mediated by circulating Ig (GO:0002455), Ig-mediated immune response (GO:00160664), and B-cell-mediated immunity (GO:0019724), highlighting the activation of the humoral immune response observed in the more advanced stages of clinical PTB. C2 and C4A expression has been associated with the pathogenesis of human diseases caused by *Bordetella pertussis* (bta:05133) and *Staphylococcus aureus* infections (bta:05150). These findings are supported by the discovery that changes in the AS of C4A were increased when *Staphylococcus aureus* mastitis developed (53). Furthermore, components of the complement system, such as C1S, C2, C3, C4A, C6, C7, and C8 genes, were differentially expressed and underwent AS in mammary gland tissues of cows naturally infected with *Staphylococcus aureus* (31). Interestingly, the bovine *C4A* gene is closely linked to the MHC class II region on *Bos taurus* chromosome 23, which shows a significant association with susceptibility to intramammary infections (54, 55). In the comparison of cows with diffuse lesions *vs.* focal lesions, three alternatively spliced genes (*IFI30*, *PSAP*, and *ASAH1*) involved in the lysis of vacuoles by lysosomes and MHC class II-restricted antigen processing were upregulated. Altogether, our data identify potential mechanistic biomarkers and novel opportunities for the development of splicing-based therapies for inflammatory and autoimmune diseases.

## 5 Conclusion

Earlier studies have focused on the role of transcription in setting up the host response against pathogens. In this study, we addressed the underexplored role of AS in regulating gene expression and the host immune response in MAP-infected cattle. This wide analysis of AS events is still rare in human research, and even more so in a veterinary context. Furthermore, our study seeks to explore differences in alternative splicing events taking place in immune-associated pathways that could be paralleled to human inflammatory and infectious diseases. Our results demonstrated that MAP infection causes changes in AS in cows with different PTB outcomes. In the PB of cows with focal lesions, alternatively spliced genes involved in the endocytosis and recognition of MAP antigens might contribute to immune tolerance to MAP antigens, as observed in EBV and Kaposi sarcoma-associated herpesvirus infections in humans. In the PB samples of cows with diffuse lesions *vs.* controls, the AS of genes involved in neutrophil degranulation and platelet activation may simultaneously control MAP growth and facilitate granuloma formation. As the infection progresses to a clinical stage, AS of genes involved in the lysis of MAP-containing phagosomes might lead to apoptosis and disruption of the intestinal mucus layer by similar mechanisms as seen in humans infected with *Entamoeba histolytica*. In ICV samples of cattle with diffuse lesions *vs*. focal lesions, AS and the upregulation of genes involved in endocytosis and antigen processing and presentation might activate an uncontrolled pro-inflammatory response characteristic of the clinical disease. Uncontrolled antigen presentation and complement activation have also been associated with several inflammatory and autoimmune human diseases. Overall, our results identify the AS of important immune genes as a mechanistic link between PTB in cattle and many inflammatory and autoimmune human diseases and provide important clues for future therapies in human pathologies.

## Data availability statement

RNA-Seq raw data have been deposited in the NCBI Gene Expression Omnibus (GEO) database under the accession number GSE137395. The datasets generated during the current study are available from the corresponding author on reasonable request.

## Ethics statement

The animal study was approved by The Animal Ethics Committee of the Servicio Regional de Investigation y Desarrollo Agroalimentario (SERIDA). The study was conducted in accordance with the local legislation and institutional requirements.

## Author contributions

GB: Formal analysis, Investigation, Methodology, Validation, Visualization, Writing – original draft. JL: Conceptualization, Data curation, Formal analysis, Investigation, Methodology, Resources, Software, Supervision, Writing – review & editing. RC: Methodology, Resources, Writing – review & editing. MA: Conceptualization, Formal analysis, Funding acquisition, Investigation, Methodology, Project administration, Resources, Supervision, Visualization, Writing – review & editing.
