# Corrigendum: Alternative splicing of pre-mRNA modulates the immune response in Holstein cattle naturally infected with *Mycobacterium avium* subsp. *paratuberculosis*

**DOI:** 10.3389/fimmu.2024.1430718

**Published:** 2024-05-29

**Authors:** Gerard Badia-Bringué, José Luis Lavín, Rosa Casais, Marta Alonso-Hearn

**Affiliations:** ^1^ Department of Animal Health, NEIKER-Basque Institute for Agricultural Research and Development, Basque Research and Technology Alliance (BRTA), Derio, Bizkaia, Spain; ^2^ Universidad del País Vasco/Euskal Herriko Unibertsitatea (UPV/EHU), Leioa, Bizkaia, Spain; ^3^ Department of Applied Mathematics, NEIKER- Basque Institute for Agricultural Research and Development, Basque Research and Technology Alliance (BRTA), Derio, Bizkaia, Spain; ^4^ Center of Animal Biotechnology, Servicio Regional de Investigación y Desarrollo Agroalimentario (SERIDA), Deva, Spain

**Keywords:** paratuberculosis, alternative splicing, chronic inflammatory diseases, autoimmune diseases, molecular mechanism

In the published article, there was an error regarding the affiliation(s) for Gerard Badia-Bringué. As well as having affiliation 1 (Department of Animal Health, NEIKER-Basque Institute for Agricultural Research and Development, Basque Research and Technology Alliance (BRTA), Derio, Bizkaia, Spain), he should also have affiliation 2 (Universidad del País Vasco/Euskal Herriko Unibertsitatea (UPV/EHU), Leioa, Bizkaia, Spain).

The authors apologize for this error and state that this does not change the scientific conclusions of the article in any way. The original article has been updated.

